# The Impact of Humanistic Postoperative Care Integrated With the 6S Management Model on Postoperative Delirium and Psychiatric Symptoms in Patients With Schizophrenia Undergoing Surgery

**DOI:** 10.62641/aep.v54i3.2239

**Published:** 2026-06-15

**Authors:** Junxia Tong, Meijuan Lan, Yanling Xiao, Xiaoli Liu

**Affiliations:** ^1^Department of Anesthesiology and Surgery, Yongkang First People’s Hospital of Wenzhou Medical University, 321300 Jinhua, Zhejiang, China; ^2^Department of Nursing, People’s Hospital of Pingyang, 325400 Wenzhou, Zhejiang, China; ^3^Department of Breast and Thyroid Surgery, The First Affiliated Hospital of Wenzhou Medical University, 325000 Wenzhou, Zhejiang, China; ^4^Department of Nursing, The People’s Hospital of Cangnan, 325800 Wenzhou, Zhejiang, China

**Keywords:** 6S management model, humanistic postoperative care, schizophrenia, postoperative delirium, stress response, psychiatric symptoms

## Abstract

**Background:**

To explore the effects of humanistic postoperative nursing integrated with the 6S management model on psychiatric symptoms, postoperative delirium and stress response in patients with schizophrenia undergoing surgery.

**Methods:**

This study adopted a retrospective research design, selecting 80 schizophrenia patients who underwent elective general surgery treatment at People’s Hospital of Pingyang between 1 August 2023 and 1 August 2025 as the research subjects. Based on the different treatment methods, the study subjects were divided into a control group (n = 40, receiving routine care) and an observation group (n = 40, receiving humanistic postoperative care integrating the 6S management model on the basis of routine care). Statistical analysis was used to examine the incidence of postoperative delirium, incidence of complications, stress response levels before and after nursing care, social function scores, Positive and Negative Syndrome Scale (PANSS) scores, and mental status scores in both groups.

**Results:**

The incidence of postoperative delirium and complications was lower in the observation group than in the control group. There were no significant differences in stress response levels, social function scores, PANSS scores, and mental status scores between the two groups before nursing care (*p* > 0.05). After nursing care, the observation group had lower stress response levels, PANSS scores, mental status scores and higher social function scores than the control group (*p* < 0.001).

**Conclusions:**

Humanistic postoperative nursing integrated with the 6S management model can reduce the incidence of postoperative delirium, complications, alleviate stress response, improve social function, and reduce psychiatric symptoms in patients with schizophrenia undergoing surgery.

## Introduction

Schizophrenia is a mental illness with a high relapse rate and a long course. The specific causes of the disease are not yet fully understood [1, 2]. Schizophrenia patients commonly experience thought disorders, emotional disorders, and perceptual disorders. Once surgery is required, the difficulty of clinical treatment and nursing will increase [3, 4]. Surveys show that postoperative nursing care for schizophrenia patients undergoing surgery faces many difficulties, such as large fluctuations in patients’ emotions and unclear cognition, leading to poor nursing compliance and ultimately hindering postoperative recovery [5, 6]. Routine nursing measures used for schizophrenia patients undergoing surgery cannot significantly improve patients’ stress response and quality of life, and have significant limitations [7, 8]. The 6S management model originated from on-site management in Japanese enterprises. Its core elements include sorting, straightening, sweeping, cleaning, self-discipline, and safety. Through standardized and regulated processes, it optimizes on-site management and enhances work efficiency and quality [9]. Due to its simplicity in operation and remarkable effectiveness, this model has been widely applied in the medical care field, especially in the management of operating rooms and the optimization of postoperative care processes, providing new practical ideas for perioperative care in psychiatry. The 6S management model was initially used in enterprise management and has been widely used in the field of medical and nursing services due to its advantages such as simple operation and significant effects. Humanistic postoperative care emphasizes respecting patients’ values, preferences, and unique experiences, promoting collaborative decision-making, and creating a supportive and predictable care environment [10]. Combining humanistic postoperative care with the 6S management model may produce synergistic effects. Therefore, this study aims to explore the impact of this comprehensive care model on patients undergoing surgery for schizophrenia, providing a feasible and effective non-pharmacological care strategy for clinical practice.

## Materials and Methods

### General Information

This study adopted a retrospective research design, selecting 80 schizophrenia patients who underwent surgical elective general surgery treatment at People’s Hospital of Pingyang with the ethical approval number of LW-2026-012 between 1 August 2023 and 1 August 2025.This study adhered to the relevant ethical requirements of the Declaration of Helsinki. All patients and their families signed informed consent forms related to the surgery and care. All clinical data of the included patients were uniformly extracted from the electronic and paper medical records of the hospital by two specially trained researchers. Before extraction, a unified data extraction form was developed, and the standards for extracting each item of data were clearly defined. During the extraction process, a double-check method was adopted, and after extraction, the consistency was verified through Kappa test (Kappa = 0.92) to strictly control the bias in data extraction. Both groups consisted of the same team of 10 nurses. The subjects were divided into a control group and an observation group, with 40 cases in each group, depending on the treatment method.

Inclusion criteria: (1) meeting the diagnostic criteria for schizophrenia [11]; (2) undergoing surgical treatment in the research hospitals during the study period; (3) complete medical records available for review; (4) being over 18 years old.

Exclusion criteria: (1) patients with severe liver and kidney dysfunction; (2) patients with a history of drug dependence; (3) patients with coagulation disorders; (4) patients with systemic infectious diseases.

### Methods

The nursing care for both groups of patients lasted from the day of surgery until discharge, with an average duration of 12.5 ± 3.2 days. During the study period, all patients continued to take the regular antipsychotic drugs prescribed by their psychiatrists, and the types or doses of antipsychotic drugs were not changed during the postoperative hospitalization. In this study, the length of hospital stay was included as a confounding variable in the statistical analysis for correction. Meanwhile, a balance analysis was conducted on potential confounding factors such as the type of surgery, anesthesia method, perioperative analgesic/sedative drug use, and electrolyte levels for both groups of patients to ensure no significant differences between the groups (*p* > 0.05).

#### Control Group

Conventional care was provided, and all care measures were strictly carried out in accordance with the department’s nursing standards. All operations were conducted at a fixed frequency every day. Psychotherapy sessions are held twice a week, each lasting 45 minutes. Health education sessions are held daily, each lasting 20 minutes. The specific measures are as follows: (1) Before surgery, assess the patient’s level of consciousness, nutritional status, sleep status, heart rate, and blood pressure. One day before surgery, understand the changes in the patient’s condition, grasp the patient’s living habits, focus on assessing the patient’s psychological state, and inform the patient of the importance of maintaining a peaceful mindset for the smooth conduct of surgery; urge the patient to maintain good personal hygiene and carry out preoperative training, continuously monitor the patient’s medication, and prevent the patient from arbitrarily increasing or decreasing the dosage of medication; (2) Invite family members to actively participate in psychological care work and provide the patient with high psychological support; correct the patient’s misconceptions and enable them to maintain a certain level of confidence in the surgery; assist the patient in completing routine blood and urine tests before surgery, and psychiatrists need to participate in the entire surgical process and properly prepare restraints to prevent accidents; (3) Before the patient enters the operating room, carefully verify the patient’s basic information. After confirming that everything is correct, establish an effective intravenous access and ensure that all instruments and equipment are functioning properly. During the operation, continuously monitor the patient’s heart rate and body temperature, and actively cooperate with the doctor in passing surgical instruments. Continuously monitor the patient’s facial reactions during the operation. If agitation occurs, inform the surgeon immediately. The anesthesiologist and psychiatrist will consult together to increase the dosage of anesthesia to ensure the patient can undergo the operation smoothly. Use protective restraints appropriately during the operation to prevent the patient from engaging in suicidal, self-harm, or harmful behavior towards others. The purpose of using protective restraints must be explained to the patient and their family before the operation. (4) Postoperatively, assist the patient to maintain a supine position for 6 hours. After the anesthesia wears off, assist them to maintain a semi-recumbent position. After passing gas, transition from liquid to semi-liquid diet, and then to regular food. Encourage the patient to get out of bed and move around as soon as possible, explaining in detail the dangers of prolonged bed rest or excessive inactivity. Monitor the patient’s blood pressure and heart rate every 2 hours postoperatively, and record the patient’s 24-hour fluid intake and output in detail. Carefully clean any residual secretions from the mouth and nasal cavity, and perform regular skin care for the patient. For patients with indwelling urinary catheters, ensure unobstructed drainage and record the color and amount of drainage fluid in detail. (5) After the anesthesia wears off, patients generally feel pain of varying degrees. Nurses should inform patients that pain is a normal phenomenon. At the same time, they should reasonably assess the degree of pain. For mild pain, music therapy or deep breathing therapy should be used, and for moderate to severe pain, medication should be used for analgesia. (6) Encourage patients to do a good job of self-monitoring, carry out health education activities on hygiene knowledge in a timely manner, encourage patients to actively participate in social activities, and further improve patients’ social function and mental state. Let patients and their families fully realize the harm of disease recurrence and guide patients on how to identify the signs of disease recurrence.

#### Observation Group

On the basis of the control group, a postoperative care method integrating the 6S management model and humanistic care was applied. Humanistic care-related treatment measures are performed three times a day, each time for 15–20 minutes. The 6S management framework was reorganized, and humanistic care was integrated into each element, forming a comprehensive approach rather than a simple combination: (1) Forming a nursing team. All 10 nurses were trained and mastered the 6S management model and the concept of humanistic nursing, and the training focused on the integration points and implementation skills of humanistic care in each link of 6S management. After passing the training, they were put into their posts. (2) Sorting. Distinguish between necessary and non-necessary items, remove surgical items that have not been used for a long time in the operating room, provide sufficient space for surgical operations, reduce the interference of the environment on medical staff, make it convenient for medical staff to take and use them, and arrange the daily necessities of patients in a fixed and easy-to-reach position according to their living habits and physical conditions, reflecting humanistic care; (3) Straightening. Accurately locate surgical items and mark their names, prices, etc., so that medical staff can find them; check and maintain surgical items in a timely manner to ensure that they are usable; establish a personalized nursing file for each patient, record their mental state, living habits, surgical conditions and nursing needs in detail, and realize the accurate and targeted humanistic nursing; (4) Cleaning. Maintain the cleanliness of the operating room environment to prevent cross-infection of patients; record in detail the usage time and maintenance time of infusion equipment and electrocardiogram monitors to ensure that disinfection work is in place; adjust the ward environment according to the patient’s sensory preferences, such as adjusting the light and sound volume, keeping the ward quiet and comfortable, and reducing the sensory stimulation of patients with schizophrenia; (5) Standardizing. Formulate equipment maintenance system, cleaning and disinfection system and item placement system according to the actual situation of the department, implement a supervision mechanism of mutual inspection by groups, self-inspection by individuals and random inspection by head nurses, and rectify the problems that have occurred within a time limit; formulate a standardized humanistic nursing process, including standardized psychological communication language, personalized pain care plan, and standardized cognitive training process, to ensure the standardization and consistency of humanistic care implementation; (6) Professionalism. Conduct 6S management training, requiring each nurse to be able to accurately identify commonly used surgical items and master emergency handling skills and master the communication skills with schizophrenia patients, psychological care methods and cognitive training skills; require nurses to use polite language with patients and maintain a high sense of responsibility and patience; conduct regular assessments and incorporate assessment results into performance evaluation; (7) Safety. Conduct regular safety education for nurses to enhance their risk prevention awareness, prevent medication errors or occupational injuries, and ensure the safety of medical staff and patients; strengthen the psychological safety protection of patients, timely identify and intervene the suicidal and self-harm tendencies of patients, and establish a two-way communication channel between patients, families and medical staff to eliminate the psychological anxiety of patients; (8) Humanistic postoperative care embedded in the 6S management framework. Educate patients and their families about disease-related knowledge, organize special meetings on psychological issues, and patiently answer patients’ questions; create a comfortable ward environment for patients, and try to avoid sound and light stimulation at night to prevent sleep disturbances; communicate with patients based on their personality traits and interests to build their trust in medical staff and reduce their anxiety and fear; nurses should consider issues from the patient’s perspective, fully respect their privacy, and guide them to maintain an optimistic attitude; correct risk factors that may induce delirium and ensure electrolyte balance; implement a double-checking system for medication to prevent errors; conduct cognitive training based on the patient’s cognitive level, such as attention training or memory training, encourage patients to actively participate in social activities, and guide them to participate in recreational activities such as drawing, crafts, and singing, using role-playing to help them relax and experience the characteristics of different personalities; focus on explaining to patients and their families how to deal with emergencies after surgery, maintain a calm mindset when facing external pressure, and share their feelings about facing pressure with medical staff.

### Observation Indicators

The incidence of postoperative delirium, incidence of complications ((1) pressure injury, (2) surgical site infection, (3) urinary tract infection), stress response level before and after nursing (including the following indicators: (1) diastolic blood pressure, (2) systolic blood pressure, (3) heart rate), social function score (using the Scale of Social Function in Psychosis Inpatients (SSPI) scale [12]), Positive and Negative Syndrome Scale (PANSS) scores [13], and mental state score (using the Inpatient Psychiatric Rehabilitation Outcome Scale (IPROS) [14]) were analyzed in the two groups. The SSPI, PANSS and IPROS scale scores were all evaluated by two psychiatric nurses who had received professional training and passed the consistency assessment (Kappa > 0.85). The raters were blinded (unaware of the patients’ groupings) and conducted synchronous scoring one day before the nursing care and on the day of discharge (the end of the nursing care). If there was a score difference (≥2 points), a third senior psychiatric nurse would review and arbitrate to ensure the objectivity and accuracy of the scoring results. All quality control measures related to the scale scoring were standardized in advance, and the entire scoring process was recorded to avoid measurement or recording deviations (The higher the PANSS and IPROS scores, the more severe the condition of the schizophrenia surgery patients; the higher the SSPI score, the better the social function.).

(1) SSPI: This scale consists of 12 items, it is divided into three dimensions: life skills, social activity skills, and mobility and social competence, each rated on a 0–4 scale, with total scores ranging from 0 to 48. Higher scores indicate better social functioning. The scale has demonstrated good reliability and validity in patients with schizophrenia (Cronbach’s α = 0.85).

(2) PANSS: The PANSS includes 30 items, each scored from 1 (absent) to 7 (extreme), covering positive symptoms, negative symptoms, and general psychopathology. Total scores range from 30 to 210, with higher scores indicating more severe symptoms. The scale has well-established reliability and validity in schizophrenia research (Cronbach’s α = 0.87).

(3) IPROS: The IPROS consists of 36 items across five subscales: performance in occupational therapy, daily activities, socialisation, personal hygiene, and level of interest in external events. Each item is rated on a 0–4 scale, with total scores ranging from 0 to 144. Higher scores indicate worse mental status. The scale has been validated in Chinese populations with schizophrenia (Cronbach’s α = 0.97).

(4) Postoperative delirium: The Confusion Assessment Method (CAM) [15] was used for delirium screening. Trained and qualified nurses conducted evaluations twice a day, in the morning and afternoon, from the first to the seventh day after surgery. Patients who screened positive by the CAM underwent further diagnostic evaluation by a psychiatrist according to Diagnostic and Statistical Manual of Mental Disorders, Fifth Edition, Text Revision (DSM-5-TR) [16] criteria to confirm the delirium diagnosis. The incidence of postoperative delirium was calculated based on the final psychiatric diagnoses.

### Statistical Analysis

Data analysis was performed using SPSS 24.0 software (IBM Corp., Armonk, NY, USA). Quantitative data are expressed as mean ± standard deviation (SD), and differences between groups were compared using independent samples *t*-tests. For within-group comparisons of quantitative data before and after nursing care, paired samples *t*-tests were used. Categorical data are expressed as frequency and percentage (n (%)), and between-group distribution comparisons were performed using chi-square tests. If the expected frequency was less than 5, Fisher’s exact test was used for between-group comparisons. A *p*-value < 0.05 was considered statistically significant.

## Results

### Comparison of Baseline Data Between the Two Groups of Research Subjects

There were no statistically significant differences in baseline data such as gender, age, weight, duration of schizophrenia, years of education, and chlorpromazine equivalent of antipsychotic drugs between the two groups of patients (*p* > 0.05), indicating comparability. See Table 1.

**Table 1.** **Comparison of baseline data between the two groups of research subjects ($\bar{x}$ ± s/n (%))**.

Factor	Observation group (n = 40)	Control group (n = 40)	*t*/χ^2^	*p*-value
Male/Female	25 (62.50)/15 (37.50)	24 (60.00)/16 (40.00)	0.053	0.818
Age (years)	45.45 ± 6.66	45.23 ± 6.62	0.148	0.883
Weight (kg)	59.51 ± 7.36	57.02 ± 10.42	1.234	0.221
Duration (years)	7.09 ± 2.41	7.13 ± 2.32	0.076	0.940
Years of education (years)	10.23 ± 2.02	10.15 ± 2.01	0.178	0.860
Chlorpromazine equivalent (mg/d)	425.6 ± 98.3	438.2 ± 102.1	0.562	0.576

### Comparison of Postoperative Delirium and Complication Rates Between the Two Groups

The incidence of postoperative delirium in the observation group (2.50% vs. 20.00%, *p* < 0.05) and the overall complication rate (2.50% vs. 22.50%, *p* < 0.01) were significantly lower than those in the control group. Regarding specific complications, the incidence of pressure ulcers, surgical site infections, and urinary tract infections in the observation group was not statistically different from those in the control group (*p* > 0.05). See Table 2.

**Table 2.** **Comparison of postoperative delirium and complication rates between the two groups (n (%))**.

Group	Postoperative delirium incidence	Pressure injury	Surgical site infection	Urinary tract infection	Incidence of complications
Observation group (n = 40)	1 (2.50)	0 (0.00)	1 (2.50)	0 (0.00)	1 (2.50)
Control group (n = 40)	8 (20.00)	2 (5.00)	3 (7.50)	4 (10.00)	9 (22.50)
*p*-value	0.033*	0.493	0.617	0.116	0.006**

Note: *p < 0.05, **p < 0.01.

### Comparison of Stress Response Levels and Social Function Scores Between the Two Groups Before and After Nursing Care

Before nursing care, there were no significant differences between the two groups in all stress response and social function indicators. After nursing care, the observation group had significantly lower diastolic/systolic blood pressure and heart rate than the control group, and significantly higher scores in daily living skills, social skills, activity level and social interaction, and total score (all *p* < 0.001). Within each group, all indicators showed significant improvement from before to after nursing care (all *p* < 0.001). See Table 3.

**Table 3.** **Comparison of stress response levels and social function scores before and after nursing care between the two groups ($\bar{x}$ ± s)**.

Factor		Observation group (n = 40)	Control group (n = 40)	*t*-value	*p*-value
Diastolic blood pressure (mmHg)				6.496	<0.001
	Pre-care	70.25 ± 3.13	70.10 ± 3.11		
	Post-care	75.13 ± 3.15***	80.28 ± 3.86***		
Systolic blood pressure (mmHg)				9.893	<0.001
	Pre-care	106.50 ± 3.46	106.55 ± 3.53		
	Post-care	112.30 ± 3.73***	120.55 ± 3.72***		
Heart rate (beats/min)				12.852	<0.001
	Pre-care	74.23 ± 2.24	74.32 ± 2.32		
	Post-care	76.05 ± 1.57***	82.65 ± 2.79***		
Life skills				5.616	<0.001
	Pre-care	4.30 ± 1.24	4.37 ± 1.16		
	Post-care	8.92 ± 1.80***	6.76 ± 1.64***		
Social activity skills (points)				4.407	<0.001
	Pre-care	6.78 ± 1.49	6.67 ± 1.52		
	Post-care	12.15 ± 2.58***	9.76 ± 2.27***		
Mobility and social competence				7.891	<0.001
	Pre-care	8.10 ± 2.09	8.28 ± 2.09		
	Post-care	15.75 ± 2.62***	11.13 ± 2.61***		
Total score (points)				10.942	<0.001
	Pre-care	19.43 ± 3.05	19.07 ± 2.92		
	Post-care	36.82 ± 3.41***	27.65 ± 4.02***		

Note: Compared with before nursing within the same group, ***p < 0.001.

### Comparison of PANSS Scores Between the Two Groups Before and After Nursing Care

After nursing care, the observation group had significantly lower PANSS scores in positive symptoms, negative symptoms, general psychopathology, and total score compared with the control group (all *p* < 0.001). Within both groups, all PANSS scores decreased significantly from pre‑care to post‑care (all *p* < 0.001). See Table 4.

**Table 4.** **Comparison of PANSS scores between the two groups before and after nursing care ($\bar{x}$ ± s, points)**.

Factor		Observation group (n = 40)	Control group (n = 40)	*t*-value	*p*-value
Positive symptoms				5.838	<0.001
	Pre-care	22.59 ± 1.99	23.47 ± 2.26		
	Post-care	18.12 ± 1.57***	20.35 ± 1.82***		
Negative symptoms				5.681	<0.001
	Pre-care	29.30 ± 3.70	30.02 ± 2.15		
	Post-care	24.18 ± 2.13***	26.83 ± 2.04***		
General psychopathology				6.494	<0.001
	Pre-care	36.39 ± 2.90	36.59 ± 2.91		
	Post-care	28.57 ± 2.34***	32.16 ± 2.58***		
Total score (points)				9.53	<0.001
	Pre-care	88.24 ± 2.98	87.76 ± 4.38		
	Post-care	70.15 ± 4.26***	78.92 ± 3.97***		

Note: Compared with before nursing within the same group, ***p < 0.001. PANSS, Positive and Negative Syndrome Scale.

### Comparison of Mental Status Scores Before and After Nursing Care Between the Two Groups

After nursing care, the observation group had significantly lower scores than the control group in all domains of mental status, including daily activities, personal hygiene, level of interest in external events, socialisation, performance in occupational therapy, and total score (all *p* < 0.001). Within both groups, all IPROS scores decreased significantly from pre‑care to post‑care (all *p* < 0.001). See Table 5.

**Table 5.** **Comparison of mental status scores before and after nursing care between the two groups ($\bar{x}$ ± s, points)**.

Factor		Observation group (n = 40)	Control group (n = 40)	*t*-value	*p*-value
Daily activities				20.789	<0.001
	Pre-care	26.70 ± 1.81	26.80 ± 1.77		
	Post-care	12.40 ± 1.22***	18.77 ± 1.49***		
Personal hygiene				13.075	<0.001
	Pre-care	17.92 ± 1.65	17.85 ± 1.68		
	Post-care	8.87 ± 1.01***	12.42 ± 1.37***		
Level of interest in external events				9.317	<0.001
	Pre-care	22.73 ± 1.65	22.62 ± 1.58		
	Post-care	15.45 ± 1.63***	18.70 ± 1.49***		
Socialisation				29.268	<0.001
	Pre-care	19.55 ± 1.67	19.52 ± 1.66		
	Post-care	8.83 ± 1.01***	13.43 ± 1.13***		
Performance in occupational therapy				16.537	<0.001
	Pre-care	25.55 ± 1.22	25.67 ± 1.14		
	Post-care	18.22 ± 1.07***	22.32 ± 1.14***		
Total score (points)				22.894	<0.001
	Pre-care	112.63 ± 3.71	113.39 ± 3.77		
	Post-care	63.77 ± 3.89***	85.64 ± 4.58***		

Note: Compared with before nursing within the same group, ***p < 0.001.

## Discussion

Studies have shown that the social function of patients with schizophrenia is severely affected, not only impacting their own quality of life but also increasing the pressure on their families and society [17, 18, 19]. Most patients experience varying degrees of cognitive impairment in their struggle against the disease, and combined surgery exacerbates their physical and mental stress, leading to a significant decrease in their clinical compliance [3, 5]. Patients with schizophrenia themselves have neurocognitive abnormalities such as executive function deficits in the prefrontal cortex and excessive activation of the amygdala [20]. The physical stress caused by surgical trauma can further aggravate neurotransmitter imbalance and cognitive impairment through excessive activation of the hypothalamic-pituitary-adrenal (HPA) axis [21]. At the same time, the unfamiliar environment and medical procedures after surgery will amplify the core symptoms such as suspicion and fear of the patients, ultimately leading to an increased risk of delirium and complications [22]. The 6S management model reduces the risk of hallucinations in patients with schizophrenia by optimizing the environment and processes, and combined with humanistic care, it can strengthen the psychological support of patients and gradually improve their confidence. In this study, the incidence of postoperative delirium and complications in the observation group was lower than that in the control group. Within-group comparisons showed that both groups experienced postoperative complications, but the observation group had a markedly lower rate. Postoperative delirium is a sudden condition. Patients experience anxiety and loneliness due to psychological trauma or fear of using various instruments and equipment, which worsens their condition [23, 24]. Routine nursing methods fail to pay attention to changes in patients’ mental state, resulting in a high incidence of postoperative delirium [25]. Humanistic nursing care integrated with the 6S management model requires nurses to start from the psychological needs of patients and treat each patient and their family sincerely, so that patients and their families can fully trust medical staff. In addition, the humanistic nursing care plan integrated with the 6S management model respects the value of patients’ lives and creates a good environment for them, so that patients are in a pleasant state of mind and body and actively cooperate with medical staff in treatment and nursing care, thereby actively controlling the occurrence of postoperative complications. The 6S management, with “sorting, straightening, sweeping, cleaning, self-discipline, and safety” as its core, reduces environmental chaos and stimulation by standardizing the placement of items, lowers the risk of medical errors by standardizing operation procedures, and enhances risk warning through visual safety signs. It directly targets the characteristics of schizophrenia patients, such as their sensitivity to environmental changes and weak cognitive processing ability, to reduce external triggers that may induce hallucinations and impulsive behaviors. Meanwhile, the core of humanistic care is “patient-centered”, responding to negative symptoms caused by the disease, such as social withdrawal, emotional apathy, and lack of trust, through personalized communication, family-involved care, and simplified explanations of disease knowledge. This approach strengthens their social support system and alleviates the loneliness and anxiety brought about by surgery.

After nursing care, the observation group achieved more significant improvements in stress response levels, PANSS scores, social function scores and IPROS scores than the control group. Within-group comparisons confirmed that both groups showed significant improvements from baseline, with the observation group demonstrating superior outcomes. These results are attributed to the following: the nursing model integrating humanistic care with the 6S management framework falls under the category of high-quality nursing services. This method can reduce patients’ risky behaviors, enabling them to learn and identify triggers for risky behaviors, and consciously adopt healthy ways to cope with stress, ultimately reducing risky behaviors and alleviating patients’ mental symptoms and physical stress responses. Furthermore, the integrated nursing model requires nurses to have high overall competence. When performing nursing procedures, nurses not only need to strictly follow standard operating procedures but also fully consider the actual needs of patients. By understanding patients’ interests and personality traits, nurses can help patients understand surgical and disease knowledge, thereby establishing correct cognition and reducing negative emotions, and further improving their social function and mental status.

This study has several limitations. First, the retrospective design may introduce selection bias, although efforts were made to extract data systematically from medical records. Second, the single-center design and relatively small sample size may limit the generalizability of the result. Although this study controlled for confounding factors such as hospital stay and type of surgery, there are still potential uncontrolled confounding factors, such as the degree of family support for the patient and the patient’s cooperation with postoperative rehabilitation training. Future multi-center studies with balanced sample sizes are needed to confirm our findings. Third, the lack of long-term follow-up prevents assessment of durability of improvements. Future prospective studies with larger, multi-center samples and extended follow-up periods are needed to confirm these findings and explore mechanisms of action.

## Conclusions

In conclusion, humanistic postoperative care integrated with the 6S management model can reduce the incidence of postoperative delirium and complications in patients with undergoing surgery, alleviate patients’ stress response levels, improve social function, and reduce psychiatric symptoms as measured by PANSS and IPROS. It is worthy of application.

## Availability of Data and Materials

The datasets used and/or analysed during the current study were available from the corresponding authors on reasonable request.
